# Can temporal covariation and autocorrelation in demographic rates affect population dynamics in a raptor species?

**DOI:** 10.1002/ece3.6027

**Published:** 2020-02-07

**Authors:** Rémi Fay, Stephanie Michler, Jacques Laesser, Jacques Jeanmonod, Michael Schaub

**Affiliations:** ^1^ Swiss Ornithological Institute Sempach Switzerland

**Keywords:** covariance, European kestrel, *Falco tinnunculus*, prey abundance, reproduction, serial correlation, stochastic population growth rate, survival

## Abstract

Theoretical studies suggest that temporal covariation among and temporal autocorrelation within demographic rates are important features of population dynamics. Yet, empirical studies have rarely focused on temporal covariation and autocorrelation limiting our understanding of these patterns in natural populations. This lack of knowledge restrains our ability to fully understand population dynamics and to make reliable population forecasts. In order to fill this gap, we used a long‐term monitoring (15 years) of a kestrel *Falco tinnunculus* population to investigate covariation and autocorrelation in survival and reproduction at the population level and their impact on population dynamics. Using Bayesian joint analyses, we found support for positive covariation between survival and reproduction, but weak autocorrelation through time. This positive covariation was stronger in juveniles compared with adults. As expected for a specialized predator, we found that the reproductive performance was strongly related to an index of vole abundance explaining 86% of the temporal variation. This very strong relationship suggests that the temporally variable prey abundance may drive the positive covariation between survival and reproduction in this kestrel population. Simulations suggested that the observed effect size of covariation could be strong enough to affect population dynamics. More generally, positive covariation and autocorrelation have a destabilizing effect increasing substantially the temporal variability of population size.

## Graphical abstract

Empirical studies have rarely focused on temporal covariation and autocorrelation in vital rates limiting our understanding of these processes in natural populations. Our study in a kestrel population supported a positive covariation between survival and reproduction, with an age effect, but found weak evidence for temporal autocorrelation. Results suggest that prey abundance drives this positive covariation. Simulations show that the observed effect size of covariation is strong enough to increasing substantially the temporal variability of the population size.

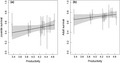

## 1 INTRODUCTION

Understanding population dynamics is a major goal for eco‐evolutionary research and the planning of conservation measures for natural populations. Most of the studies investigating population dynamics in the wild focused on the mean and the temporal variance of vital rates but ignored temporal covariation and autocorrelation structures (Fieberg & Ellner, 2001 but see Reid, Bignal, Bignal, McCracken, & Monaghan, 2004; Coulson, Gaillard, & Festa‐Bianchet, 2005; Ezard, Becker, & Coulson, 2006; Reed & Slade, 2006). Since temporal covariation among traits and temporal autocorrelation (hereafter covariation and autocorrelation) within traits can be substantial (Doak, Morris, Pfister, Kendall, & Bruna, 2005b), our understanding of population dynamics and evolutionary ecology may be incomplete (Benton & Grant, 1996; Ferson & Burgman, 1995; Ruokolainen, Lindén, Kaitala, & Fowler, 2009; Tuljapurkar, 1982; Tuljapurkar & Haridas, 2006).

Covariation among and autocorrelation within vital rates affect the stochastic population growth rate (Caswell, 2001; Tuljapurkar, 1982). Positive covariation and autocorrelation tend to decrease the stochastic growth rate and to increase the variability in population size while negative covariation and autocorrelation results in opposite patterns buffering population dynamics (Ramula & Lehtilä, 2005; Tuljapurkar, Gaillard, & Coulson, 2009). However, it is difficult to make generalization because the life‐history strategy and regime of density dependence may affect the population consequences of covariation and autocorrelation (Colchero et al., 2019; Heino & Sabadell, 2003; Paniw, Ozgul, & Salguero‐Gómez, 2018; Ruokolainen et al., 2009; Tuljapurkar et al., 2009). Covariation and autocorrelation are also critical in applied ecology issues. For instance, our ability to reliably forecast population size and to estimate extinction risks is considerably reduced when these patterns are not taken into account (Cuddington & Yodzis, 1999; Ferson & Burgman, 1995; Heino & Sabadell, 2003; Pike, Tully, Haccou, & Ferrière, 2004). Similarly temporal environmental autocorrelation could be a key factor to understand the establishment of alien species (Fey & Wieczynski, 2017).

Until now, most knowledge on this topic originates from theoretical studies based on simulated data (e.g., Cuddington & Yodzis, 1999; Heino & Sabadell, 2003; Paniw et al., 2018; Ruokolainen et al., 2009; Tuljapurkar & Haridas, 2006). Because these studies use strong simplifications, for example, assuming that environmental stochasticity transfers linearly to vital rates or that no age effect in covariance and autocorrelation patterns is present, they do not necessarily match population dynamics in the wild (van de Pol et al., 2011). Thus, describing empirically covariation and autocorrelation in vital rates is critical to assess the effect of these patterns on population dynamics. Although survival and reproduction have been extensively studied, few empirical studies have investigated how these vital rates are autocorrelated through time and how they are correlated at population level. The few studies estimating autocorrelation found mixed evidence for their presence and reported generally a minor effect on the population growth rate and extinction risk (Silva, Raventos, Caswell, & Trevisan, 1991; Morris et al., 2011; van de Pol et al., 2011). However, these studies are by far too spares to make general statements. Studies estimating covariations among vital rates are more consistent and suggested that covariations are frequent and may impact population dynamics (Davison, Nicolè, Jacquemyn, & Tuljapurkar, 2013; Ezard et al., 2006; Reid et al., 2004; Sim, Rebecca, Ludwig, Grant, & Reid, 2011). A substantial part of the temporal variability of the population growth rate can be explained by covariation among vital rates. For instance, Coulson et al. (2005) estimated that covariation among vital rates explains between one‐third and one‐half of the variation in population growth rate of large ungulate species.

Covariation among vital rates and temporal autocorrelation at the population level can be driven by different processes including environmental variation (Knops, Koenig, & Carmen, 2007), density dependence (Sinclair & Pech, 1996), or trade‐offs (Van Tienderen, 1995). For example, survival and reproduction may be positively (or negatively) correlated if a particular environmental condition affects these two traits in the same or in opposite way, respectively (Knops et al., 2007). Similarly they would be positively or negatively autocorrelated if these environmental conditions persist over time (Fey & Wieczynski, 2017). Alternatively, survival and reproduction may be negatively correlated if there is a trade‐off between them, that is, due to reproductive costs (Stoelting, Gutierrez, Kendall, & Peery, 2014), or because one trait is compromised by the other due to negative density dependence. Negative density dependence could also lead to negative autocorrelation within traits. In case of positive density dependence (i.e., an Allee effect), opposite patterns may emerge with a positive covariation between survival and reproduction and a positive autocorrelation over time.

Patterns of covariation and autocorrelation among and within vital rates are expected to vary with individual attributes such as age or sex (Ezard et al., 2006; Reid et al., 2004). Regarding age, life‐history traits of young individuals are typically more variable than those of adults (Gaillard, Festa‐Bianchet, & Yoccoz, 1998). Owing to their lower competitiveness, lack of experience or physiological immaturity, young individuals are more sensitive to environmental fluctuations (Altwegg, Dummermuth, Anholt, & Flatt, 2005; van Oudenhove, Gauthier, & Lebreton, 2014), density dependence (Fowler, 1987) and also more likely to display life‐history trade‐offs (Tavecchia et al., 2005). Thus, we may expect stronger covariation and autocorrelation in vital rates of young individual than in those of adults.

Although it is known for a long time from theory that covariation among and autocorrelation within vital rates are important for population dynamics (Tuljapurkar, 1982; Van Tienderen, 1995), empirical ecologists have paid little attention to these patterns as well as to the underlying ecological factors which drive them (Doak, Gross, & Morris, 2005a; Fieberg & Ellner, 2001; Reed & Slade, 2006; Ruokolainen et al., 2009). For instance, while simulations have shown that the effect of temporal autocorrelation on population dynamics can depend on which vital rate is affected (Heino & Sabadell, 2003), it is not known empirically if autocorrelation patterns vary depending on vital rates and if so, to what extent these variations are consistent across species or ecological contexts. The lack of attention from empirical ecologists may be due to the advanced mathematical and statistical tools involved in this topic, for example, matrix population models and the estimation of random effects (Caswell, 2001; Kéry & Schaub, 2012; Tuljapurkar, 1982). Another reason of the limited empirical investigation of covariation and autocorrelation in vital rates is the long‐term data needed (Doak, Gross, et al., 2005a). At least 10 or 16 years of continuous monitoring on different vital rates seems required according to Swanson (1998) and Gilljam et al. (2019), respectively.

Our study attempts to fill these gaps in our current knowledge. Based on a long‐term monitoring program of a Eurasian kestrel (*Falco tinnunculus*, hereafter kestrel) population, we investigate the patterns of variation in survival and reproduction. We estimated both covariation and autocorrelation for reproduction, and juvenile (i.e., first year) and adult survival. Based on demographic simulations, we also assessed the potential effect of the observed covariation and autocorrelation values on population dynamics. As the demography of the kestrel is strongly affected by the abundance of its main prey, that is, voles (Fargallo et al., 2009; Korpimäki & Norrdahl, 1991; Laaksonen, Lyytinen, & Korpimäki, 2004), we expected fluctuations in food availability to drive covariation and autocorrelation patterns. Under the *food availability hypothesis*, we predict both juvenile and adult survival to be positively related to reproduction. Because young individuals are more sensitive to environmental fluctuations, we predict a stronger covariation between juvenile survival and reproduction than between adult survival and reproduction. Predictions for autocorrelation patterns are more difficult to formulate because they will depend on the temporal pattern in the abundance of their main prey. Vole populations may cycle over short periods, for example 3 years, as well as longer periods or not at all according to both location and time (Brommer, Pietiäinen, & Kolunen, 2002; Millon et al., 2014; Pavluvčík et al., 2015; Tkadlec & Stenseth, 2001), making prediction impossible at this point. Finally, we tested the food availability hypothesis directly by assessing the relationship between reproduction and vole abundance. Based on the literature, two other nonexclusive mechanisms leading to different predictions could be considered. Under the *density dependence hypothesis*, we predict reproduction and survival to be negatively related due to density‐dependent regulation expressed within a single year. However, because such density dependence affects mainly young individuals, we predict that this negative covariation is strong for juveniles but weak or absent for the adults. Under the *trade‐off hypothesis*, we predict adult survival, but not juvenile survival, to be negatively related to reproduction due to reproductive costs. These alternative hypotheses do not predict specific patterns of autocorrelation.

## 2 MATERIAL AND METHODS

### 2.1 Study species

The kestrel is a small raptor species that is widespread in open landscapes throughout the Palearctic. In Switzerland, kestrels are found throughout the country from lowland agricultural landscapes to alpine grass‐lands up to 2,000 m altitude. Although kestrels are opportunistic foragers, voles (*Microtus* sp.) are generally the most important prey (Casagrande, Nieder, Minin, Fata, & Csermely, 2008; Korpimäki, 1986; Village, 1982). The kestrel is a nonobligate hole nester. It can breed in various locations such as disused stick‐nests of larger bird species, cliffs, tree cavities, anthropogenic structures, and nest boxes (Village, 1990). Kestrels reproduce usually once a year between April and July. The female lays a clutch of 4–6 eggs that is incubated for 30 days. The chicks leave the nest at around 30 days after hatching and are guided by both parents for another 2–4 weeks (Village, 1990). Some individuals start to reproduce when they are 1 year old, but a large proportion is assumed to start reproductive life only when 2 years old (Village, 1990).

### 2.2 Study area and data collection

The study was carried out on a population of kestrels breeding in nest boxes in Switzerland. Since 2002, a large‐scale monitoring project of nest boxes has been implemented from the west (Geneva) to the north‐east (St. Gallen) of the lowlands of Switzerland (Figure S1). Apart from urban and forest habitats, the study area is dominated by open agricultural landscape with a mix of crops and intensively farmed grassland.

From 2002 to 2016, volunteers monitored the nest boxes during the breeding season collecting fecundity and capture‐recapture data. A total of 6,187 broods were monitored allowing the estimation of productivity, that is, the number of chicks reaching the ringing age in a successful brood. Chicks were ringed with an aluminum ring at a variable, but minimal age of 15 days (*n* = 28,658). In addition, a total of 808 adults were captured with so called Bal‐chatri traps or with a scoop net at the nest box entrance. Kestrels first captured as adults were marked with an aluminum ring and in some cases with an additional alphanumeric color ring. Reencounters were either resightings (*n* = 418 individuals in total) or dead recoveries (*n* = 636). From 2007 to 2018, prey remains in nest boxes have been quantified in a part of the study area (Figure S1). The total number of voles, that is, *Microtus sp.*, and birds found per year in nest boxes were recorded. These data were used to estimate the relative abundance of voles in the kestrel diet which is known to mirror the relative densities of the voles in the field (Korpimäki, 1986; Village, 1982).

### 2.3 Estimation of vital rates, covariation, and autocorrelation

We used the Bayesian model of data analysis because it allowed the joint modeling of different data sets and the estimation of random effects in a straightforward way (Kéry & Schaub, 2012). As a starting point, we modeled productivity using the normal distribution: ${\text{Prod}}_{i,t}∼N\left(\delta_{t},\sigma_{\text{PR}}^{2}\right)$ where ${\text{Prod}}_{i,t}$ is the productivity of pair *i* in year *t*, $\delta_{t}$ is the average productivity in year *t* and $\sigma_{\text{PR}}^{2}$ is the residual variance. To estimate juvenile (i.e., annual survival from fledging to the age of 1 year), and adult survival (i.e., annual survival after the age of 1 year), we analyzed capture‐recapture‐recovery data with a multistate mark‐recapture model (Brownie, Hines, Nichols, Pollock, & Hestbeck, 1993; Kéry & Schaub, 2012). We used 6 states to control for age (two age classes), mark type (aluminum ring or aluminum and color ring) and to include recovery information. The data are summarized in an m‐array table (Lebreton, Burnham, Clobert, & Anderson, 1992), whose numbers (m) follow a multinomial distribution with cell probabilities that are a function of age‐specific survival ($\Phi^{\text{juv}},\Phi^{\text{ad}}$), recapture (*p*), and recovery probabilities (*r*). Recapture and recovery probabilities were modeled with random time effects. More details of this model are given in Appendix S1.

To estimate covariation among vital rates, we performed joint analyses meaning that a joint likelihood was formulated which ensures that the uncertainties in the estimated quantities are fully taken into account. We used two complementary approaches. First, we used a multivariate normal distribution to explicitly modeling the relationship among vital rates:

$$X∼N\left(M,\Omega \right)$$

where *X* is a matrix including the annual estimates of vital rates

$$X=[\text{logit}\left(\Phi_{t}^{\text{juv}}\right),\text{logit}\left(\Phi_{t}^{\text{ad}}\right),\log\left(\delta_{t}\right)],$$

*Μ* is a vector with the means of the vital rates $M=[\mu_{\Phi^{\text{juv}}},\mu_{\Phi^{\text{ad}}},\mu_{\delta }]$ and Ω is the variance‐covariance matrix $\Omega =\left[\begin{array}{ccc} \sigma_{\Phi^{\text{juv}}}^{2} & {\text{cov}}_{\Phi^{\text{juv}},\Phi^{\text{ad}}} & {\text{cov}}_{\Phi^{\text{juv}},\delta }\\ \ldots & \sigma_{\Phi^{\text{ad}}}^{2} & {\text{cov}}_{\Phi^{\text{ad}},\delta }\\ \ldots & \ldots & \sigma_{\delta }^{2} \end{array}\right]$.

The correlation between rates is calculated as:

$\text{Cor}\left(A,B\right)=\frac{{\text{cov}}_{A,B}}{\sigma_{A}*\sigma_{B}}$ where *A* and *B* are two different vital rates.

More details are given in Appendix S2. The annual variation in vital rates could undergo significant shrinkage in this model, that is, estimates are pulled toward the mean, since their annual variations are modeled with random time effects. The amount of shrinkage generally depends on the true process variation and the sample size (Burnham & White, 2002). In our case, shrinkage could be severe especially for survival since data are relatively sparse being dominated by dead recoveries. Because we focus more on the variability rather than on the mean of the demographic rates, we used also a second analysis based on linear regressions. In this approach, the productivity was still modeled with a random time effect but juvenile and adult survivals were expressed as a linear regression of the estimated productivity:

$$\text{logit}\left(\Phi_{t}^{a}\right)=\beta_{0}+\beta_{1}*\delta_{t}+\varepsilon_{t}^{\Phi }$$

Here, $\Phi_{t}^{a}$ is the survival probability at age *a* in year *t*, $\beta_{0}$ is the intercept, $\beta_{1}$ the slope describing the relationship between estimated productivity ($\delta_{t}$) and survival in year *t* and $\varepsilon_{t}^{\Phi }$ is the residual term that we assumed to be normally distributed with mean 0 and variance $\sigma_{\Phi }^{2}$.

To estimate the 1‐year lag temporal autocorrelation within each vital rate, we again used two complementary approaches. First, we used a residual decomposition technique (Johnson & Hoeting, 2003) in which each vital rate was modeled with random time effects:

$$\text{logit}\left(\Psi_{t}\right)=\mu_{\Psi }+\varepsilon_{t}^{\Psi }$$

where Ψ is a given vital rate, $\mu_{\Psi }$ is its mean and $\varepsilon_{t}^{\Psi }$ is the deviation of the rate in year *t* from the overall mean.

Then, we expressed the deviation in a given year as a linear effect of the deviance of a previous year:

$$\varepsilon_{t}^{\Psi }=\alpha^{\Psi }*\varepsilon_{t-1}^{\Psi }+\varepsilon_{t}^{\varepsilon }$$

where $\alpha^{\Psi }$ is the autocorrelation coefficient for the vital rate Ψ and $\varepsilon_{t}^{\varepsilon }$ is the residual that we assumed to be normally distributed with mean 0 and variance $\sigma_{\varepsilon }^{2}$.

As previously, the annual variation of vital rates could undergo significant shrinkage since their annual variations are modeled with random time effects. We relaxed this constraint in a second analysis where we modeled each trait with a fixed effect of time. Autocorrelation was then estimated directly as the correlation between $\Psi_{t}$ and $\Psi_{t+1}$ where Ψ is a given vital rate. Because arithmetic means are sensitive to extreme values, we investigated the robustness of the results by re‐estimation of the autocorrelation after removing each pair of years one by one. Autocorrelation means and their credible intervals were computed based on 6,000 replicates extracted from the posterior distributions.

### 2.4 Relationship between productivity and vole abundance

We assessed the link between productivity and vole abundance using data from the part of the study area where vole data have been collected (Figure S1). As shown above, we modeled productivity using a normal distribution: ${\text{Prod}}_{i,t}∼N\left(\delta_{t},\sigma_{\text{Prod}}^{2}\right)$ where ${\text{Prod}}_{i,t}$ is the productivity of pair *i* in year *t*, $\delta_{t}$ is the average productivity in year *t* and $\sigma_{\text{Prod}}^{2}$ is the residual variance. To estimate an index of vole abundance, we considered the annual proportion of voles in all prey remains including voles and birds. Voles are the preferred prey whereas birds are more opportunistically hunted and mostly when vole abundance is low since they are more difficult to catch (Village, 1990). Thus, we used relative abundance of voles in prey remains as an index of the annual vole abundance. We modeled the number of voles in prey remains using a binomial model: $N{\text{.vole}}_{t}∼\text{Bin}\left(\gamma_{t},N{\text{.prey}}_{t}\right)$ where $N{\text{.vole}}_{t}$ is the number of voles counted in prey remains in year *t*, $N{\text{.prey}}_{t}$ is the total number of prey remains counted in year *t*, and $\gamma_{t}$ is the estimated index of vole abundance in year *t*. Finally, the relationship between productivity and estimated vole abundance is tested in a joint linear model:

$$\log\left(\delta_{t}\right)=\beta_{0}+\beta_{1}*\gamma_{t}+\varepsilon_{t}^{\delta }$$

where $\delta_{t}$ is the average productivity in year *t*, $\beta_{0}$ is the intercept, $\beta_{1}$ the slope describing the relationship between productivity ($\delta_{t}$) and the index of the vole abundance $\left(\gamma_{t}\right)$ in year *t* and $\varepsilon_{t}^{\delta }$ is the residual term that we assumed to be normally distributed with mean 0 and variance $\sigma_{\delta }^{2}$. Capture‐recapture data from this part of the study area were too sparse to assess the relationship between kestrel survival and vole abundance.

### 2.5 Model implementation

We used the Bayesian approach for inference and Markov chain Monte Carlo (MCMC) simulation for parameter estimation. We specified vague prior distributions for all parameters. We used uniform distributions on the interval [0, 1] as priors for survival and recapture probabilities, a uniform distribution on the interval [0, 10] for productivity, a uniform distribution between 0 and 10 for the standard deviations of the temporal random effects, uniform distributions on the intervals [0, 5] and [0, 10] for variance parameters of survival and productivity, respectively, uniform distributions on the interval [−1, 1] for correlation parameters and normal distributions with mean 0 and large variance 10^3^ for regression parameters. The analyses were conducted in JAGS (Plummer, 2003) via the R package jagsUI (Kellner, 2016). Posterior summaries from three Markov chain Monte Carlo (MCMC) chains were based on 50,000 iterations after a burn‐in of 20,000 and a thinning interval of 10. We confirmed parameter convergence using the Gelman–Rubin statistic. All the R‐hat values were below 1.1 suggesting convergence of the Markov chains. We generally report the posterior means and the 95% credible intervals. We checked the fit of the capture‐recapture‐recovery model using program U‐CARE (Choquet, Lebreton, Gimenez, Reboulet, & Pradel, 2009), and we found no lack of fit (*χ* ^2^ = 48.5, *p* = .61).

### 2.6 Population simulation

With the intention of favoring biological over statistical significance (Yoccoz, 1991), we used simulation to investigate the potential effect of the estimated covariation and autocorrelation on population dynamics. We built a female‐based population model based on a prebreeding census (Caswell, 2001). The expected number of females present in year *t* + 1 is given by.

(1) $$N_{t+1}=N_{t}*\Omega \Phi_{t}^{\text{juv}}\delta_{t}+N_{t}*\Phi_{t}^{\text{ad}}$$

where $N_{t}$ is the number of adult females in year *t*, $\Phi_{t}^{\text{juv}}$ and $\Phi_{t}^{\text{ad}}$ are the survival probabilities of juveniles and adults between years *t* and *t* + 1 and $\delta_{t}$ is the annual productivity. As there was no data to estimate recruitment and adult breeding probabilities, we used the scaling parameter Ω to control for these nonestimable parameters. We fixed the value of the scaling parameter to obtain a stable population given observed averages of survival and productivity.

We parameterized this model according to 4 different scenarios: (1) no covariation and no autocorrelation, (2) covariation only, (3) autocorrelation only, and (4) both covariation and autocorrelation. As our aim was to investigate the potential effect of covariation and autocorrelation on population dynamics, we used the minima and maxima of the posterior means that we obtained from the different approaches to analyze the data (see section *Estimation of vital rates, covariation, and autocorrelation*). We generated annual productivity and survival values including or excluding covariation and autocorrelation (4 scenarios) based on the estimated minimal and maximal values of the means resulting in 8 sets of values (see Appendix S3 for a detailed description). From an initial population size of 1,000 individuals, we calculated the population trajectory for 50 years based on the population model (Equation 1). Finally, we replicated this procedure 5,000 times and calculated for each run the stochastic population growth rate, that is, $\lambda_{s}=\log{\left(\frac{N_{t}}{N_{0}}\right)}^{1/t}$ and the coefficient of variation of population sizes across the 50 years as $\text{CV}=\frac{\sigma_{N}}{\mu_{N}}$. We assessed the effect of estimated covariation and autocorrelation on the dynamics of the simulated population by comparing the means of the stochastic population growth rates and of the coefficient of variations of population sizes.

## 3 RESULTS

### 3.1 Temporal covariation between vital rates

**Figure 1:**
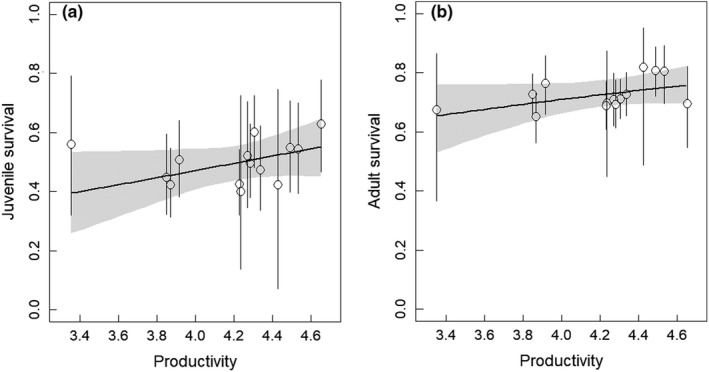
Relationship between productivity and annual survival of juvenile (a) and adult (b) kestrels. Open circles show survival estimates (±95% CRI) obtained from a model with a fixed time effect and bold lines show the survival estimates based on a linear function of productivity. For juvenile survival, we show the slope obtained after integrating climatic condition as an additional explanatory variable

Using the multivariate normal distribution, the estimates of correlation between productivity and survival of both age classes were positive (juveniles: 0.39 [−0.76, 0.98]; adults: 0.18 [−0.53, 0.83]), but the credible intervals were wide and included zero. As predicted, covariation between survival and productivity tended to be higher for juveniles than for adults. Estimated correlation between juvenile and adult survival was very small and centered on 0 (0.01, [−0.08, 0.11]). The examination of the correlation among vital rates using regression suggested also a positive relationship between productivity and survival of both age classes (Table 1). A closer graphical examination indicated an exceptional year (2013) when productivity was very low while survival was high especially for juveniles (Figure 1). These estimates can be explained by the delay of the mowing period due to exceptional wet conditions during spring in year 2013 which decreased prey accessibility during the breeding season, but not after the fledging period (Appendix S4, Figure S2). The use of a student instead of a normal distribution for the linear regression resulted in similar estimates suggesting that the outlier is too extreme to be properly accounted for in this way. However, after integrating climatic condition presented in Figure S2 as an explanatory variable or after removing this outlier, we found stronger support for a positive linear relationship between juvenile survival and productivity (slopes = 0.51 [−0.20, 1.20] and 0.66 [−0.11, 1.42], *p *(slope) > 0 = 0.93 and 0.96, respectively). In contrast to juvenile survival, the regression slope for adult survival was less affected by year 2013 (Table 1). Consistently with the first approach, the relationship between productivity and juvenile survival appeared to be stronger than that between productivity and adult survival.

**Table 1:** Estimates of the slopes of the regression of productivity against juvenile and adult survival in a kestrel population

Survival	Slope	[95% CRI]	*p* (slope) > 0
Standard linear relationship			
Juvenile	0.35	[−0.30, 0.95]	.87
Adult	0.39	[−0.16, 1.00]	.92
Linear relationship controlling for climatic condition			
Juvenile	0.51	[−0.20, 1.20]	.93
Adult	0.32	[−0.33, 0.97]	.85
Linear relationship without year 2013			
Juvenile	0.66	[−0.11, 1.42]	.96
Adult	0.39	[−0.38, 1.23]	.86

### 3.2 Temporal autocorrelation within vital rates

Estimates obtained by the residual decomposition technique suggest that both juvenile and adult survival were positively autocorrelated across time (Table 2). In contrast, productivity showed no autocorrelation with an estimate clearly centered on zero. The results of the second approach, where each vital rate was modeled with a fixed effect of time, were qualitatively similar for juvenile and adult survival, but productivity now appeared to be negatively autocorrelated across time (−0.16, 95% CRI [−0.30, −0.03]). However, this result might be driven by the pair of years 2012–2013 which were extreme (Figures S3 and S4). When autocorrelation of productivity is estimated ignoring this pair of years, the estimate became positive. The graphical examination provided little support neither for positive nor for negative autocorrelation (Figure S4) and thus we considered that the result regarding productivity is equivocal. More generally it seems that patterns of autocorrelation were less clear than patterns of covariation. Contrary to covariation, the probability that estimates of autocorrelation were positive never exceeded 0.82 regardless of the method used.

**Table 2:** Estimates of the temporal autocorrelation for productivity, juvenile, and adult survival of kestrels and the probabilities that autocorrelation was positive

Trait	Autocorrelation [95% CRI]	*p* (autocorrelation) > 0
Residual decomposition technique		
Juvenile survival	0.28 [−0.80, 1]	.70
Adult survival	0.44 [−0.66, 1]	.82
Productivity	−0.04 [−0.66, 0.66]	.42
Productivity (without 2013)	0.53 [−0.28, 0.98]	.92
Estimate with time as a fixed effect		
Juvenile survival	0.15 [−0.43, 0.65]	.70
Adult survival	0.19 [−0.35, 0.66]	.79
Productivity	−0.16 [−0.32, 0.00]	.02
Productivity (without 2013)	0.24 [0.08, 0.39]	1

### 3.3 Relationship between productivity and vole abundance

**Figure 2:**
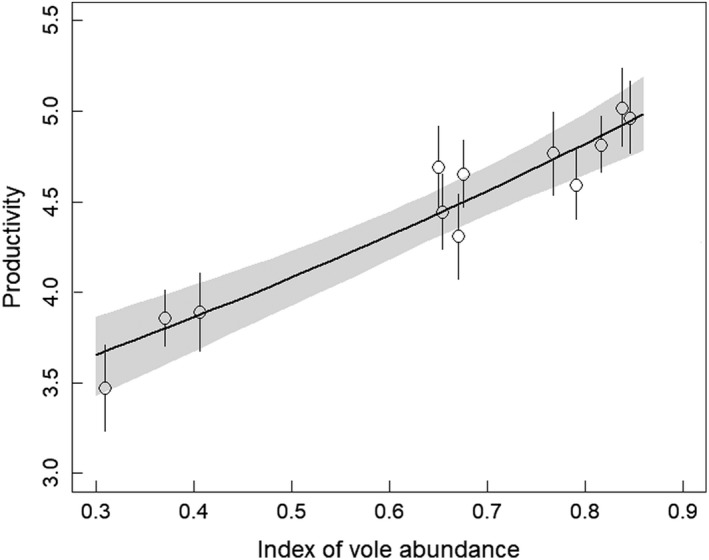
Relationship between the index of vole abundance and productivity of kestrels from 2007 to 2018. Open circles show productivity estimates (±95% CRI) obtained from a model with a fixed time effect and bold lines show the productivity estimates based on a linear function of the index of vole abundance

The proportion of voles in prey remains showed strong variation over the years from 30% to 85%. This index of vole abundance was strongly positively related to productivity (0.56 [0.40, 0.72], Figure 2) and accounted for 86% of the temporal variance of productivity. Thus vole abundance was the key factor driving kestrel reproductive process in our study area.

### 3.4 Population simulation

Using simulations we investigated the potential effect of the found levels of covariation among and autocorrelation within vital rates on population dynamics. The results presented above provide no evidence for covariation between juvenile and adult survival. Thus, we only included covariation between survival rates and productivity. Similarly, we included only autocorrelation in survival, but not autocorrelation in productivity due to the ambiguous results. The population simulations suggest that both the mean estimates of covariation and of autocorrelation were strong enough to affect population dynamics. Compared to the simulation where vital rates varied independently from each other over time and without autocorrelation, the inclusion of covariation between productivity and survival increased the variability of the population size across time by 6%–19% according to the values considered. The potential effect of autocorrelation was even stronger with an increase of the coefficient of variation of the population size by 12%–49%. When both covariation and autocorrelation were included, population size variability increased by about 13%–52%. Covariation and autocorrelation had virtually no effect on the mean of the stochastic population growth rate (Table 3).

**Table 3:** Demographic consequences of observed covariation and autocorrelation in survival and productivity in a kestrel population

	Neither covariation nor autocorrelation	Covariation only	Autocorrelation only	Covariation and autocorrelation
Minimal covariation and autocorrelation estimates				
Stochastic population growth rate				
Mean	0.00000	0.00015	−0.00011	0.00012
Population size				
CV	0.137	0.146 (+6%)	0.154 (+12%)	0.155 (+13%)
Maximal covariation and autocorrelation estimates				
Stochastic population growth rate				
Mean	0.00000	0.00040	−0.00039	0.00027
Population size				
CV	0.192	0.229 (+19%)	0.287 (+49%)	0.292 (+52%)

Note

The relative change of the coefficient of variation of the population size (CV) compared to the population simulation without covariation and autocorrelation are given in brackets. Minimal and maximal covariation and autocorrelation values are given by the mean posteriors from the different methods.

## 4 DISCUSSION

Our study in a kestrel population found evidence for positive temporal covariation between survival and productivity but less support for temporal autocorrelation in these vital rates. Results are generally consistent with the *food availability hypothesis* which is also supported by the strong positive relationship between the index of vole abundance and productivity. Furthermore, simulation results suggested that the observed effect size of covariation is strong enough to impact population dynamics, which generally became more temporally variable.

### 4.1 Temporal covariation between productivity and survival

We found support for a positive covariation between productivity and both juvenile and adult survival by both analytical methods applied. Years with high chick productivity were also years with high survival of both, juveniles and adults. This result supports the *food availability hypothesis* suggesting that favorable environmental conditions allow high demographic performance in terms of reproduction and survival. More generally, positive covariation seems to be more frequent than negative covariation and has been documented in various organisms including plants (Horvitz & Schemske, 1995), birds (Ezard et al., 2006; Reid et al., 2004; Sæther & Bakke, 2000), and mammals (Coulson et al., 2005; Morris et al., 2011). This finding suggests that density dependence and life‐history trade‐offs are generally less influential than environmental conditions to cause annual variation of vital rates at the population level (Coulson et al., 2005; Ezard et al., 2006; Stoelting et al., 2014; Tavecchia et al., 2005). Density dependence and environmental conditions do not affect vital rates in the same way. Environmental fluctuations typically affect vital rates over short periods of time resulting in increased variability whereas population density regulation occurs across longer time periods affecting primary the mean of the vital rates (Sinclair & Pech, 1996). However, density dependence may indirectly affect covariation and autocorrelation patterns by modulating the capability of demographic rates to track environmental fluctuations (van de Pol et al., 2011).

As expected, we found that the survival‐reproduction covariation at the population level changes according to the age with a stronger correlation in juveniles compared to adults. A similar pattern has been described in the red‐billed chough *Pyrrhocorax pyrrhocorax* where the covariation between survival and reproduction at the population level decreased progressively with age (Reid et al., 2004), and in an emperor penguins *Aptenodytes forsteri* population where juvenile, but not adult, survival was positively correlated to breeding success (Abadi, Barbraud, & Gimenez, 2017; Jenouvrier, Barbraud, Weimerskirch, & Caswell, 2009). The stronger survival‐reproduction covariation in juveniles is expected due to their higher sensitivity to the environmental conditions. Age‐specific sensitivity to environmental conditions has been described in a large variety of organisms (birds: van Oudenhove et al., 2014, mammals: Coulson et al., 2001, reptiles: Altwegg et al., 2005, plants: Horvitz & Schemske, 1995) making covariation between survival and reproduction more likely for juveniles than for adults. Thus, although age‐specific covariation patterns are still rarely reported, they could be widespread.

However, some studies suggested that the effect of age on trait covariation could be more complex. Sim et al. (2011) found that the population level covariation between survival and reproduction was negative in juveniles, but positive in adults. This shift in the sign of the survival‐reproduction covariation suggests different regulatory mechanism according to age. In this particular case, juvenile survival was mostly regulated by density dependence whereas adult survival was primarily affected by environmental conditions.

### 4.2 Prey availability as a cause for the covariation of survival and productivity

The index of vole abundance accounted for 86% of the annual variation in productivity suggesting that vole abundance is the main determinant of kestrel breeding success. This result is consistent with previous studies on kestrels showing that voles are the preferred prey and that vole abundance correlates positively with breeding success (Casagrande et al., 2008; Fargallo et al., 2009; Korpimäki, 1986). This strong relationship is also consistent with the *food availability hypothesis* to explain the pattern of covariation observed in this study. Consistently, other studies in raptors have reported bottom‐up control with both survival and reproduction being positively affected by prey abundance (Brommer et al., 2002; Millon et al., 2014). Food availability is a fundamental factor driving population dynamics. Experimental studies using food supplementation have shown that food availability may improve both reproduction and survival simultaneously in birds and mammals (Boutin, 1990). Our results suggest that the positive relationship between productivity and survival was stronger for juveniles than for adults. Fledglings in years of low productivity are probably faced with a double handicap. First, food supply is a critical factor determining fledging condition which strongly affects postfledging survival (Maness & Anderson, 2013; Merilä & Svensson, 1997; Perrig, Grüebler, Keil, & Naef‐Daenzer, 2017). Offspring raised under stressful conditions have lower fitness due to higher postfledging mortality (Lindström, 1999; Plard et al., 2015). Second, the postfledging stage, especially when parental care ceases, is a critical period during which mortality rates are usually high (Cox, Thompson, Cox, & Faaborg, 2014; Maness & Anderson, 2013; Naef‐Daenzer & Grüebler, 2016). Younger individuals generally still have poorer foraging ability which relates to higher postfledging mortality (Daunt, Afanasyev, Adam, Croxall, & Wanless, 2007; Orgeret, Weimerskirch, & Bost, 2016). This is likely accentuated when vole abundance is low.

### 4.3 Temporal autocorrelation

Temporal autocorrelation appears to be less evident than covariation. However, the posterior means of autocorrelation in survival were positive rather than negative which could be also consistent with the *food availability hypothesis*. Positive autocorrelation would suggest that the environmental conditions driving the vital rate fluctuate with a period longer than 1 year. As we and others have shown, the dynamics of vole abundance is a main driver for kestrel population dynamics. Voles often have populations cycles of several years (Brommer et al., 2002; Millon et al., 2014; Tkadlec & Stenseth, 2001) making positive autocorrelation plausible. Autocorrelation in vital rates has been much less studied than covariation in wild population (but see Morris et al., 2011; Reed & Slade, 2006; Silva et al., 1991). Theoretical models regularly assume positive autocorrelation in vital rates due to the general positive autocorrelation of environmental variation (Heino & Sabadell, 2003). However, Morris et al. (2011) who investigated 1‐year lag autocorrelation in seven primate species found both positive and negative relations according to both species and vital rate considered. How environmental stochasticity transfers into variation in vital rates is a complex issue and the common practice assuming perfect environmental tracking could be misleading (Laaksonen et al., 2004; van de Pol et al., 2011). Our understanding of temporal autocorrelation in vital rates of natural population is currently strongly limited due to scarcity of empirical studies investigating this topic (Ruokolainen et al., 2009).

### 4.4 Population dynamics consequences

Our simulations suggest that the observed strength of survival‐productivity covariation is strong enough to have important demographic consequences. The inclusion of autocorrelation in survival also affects population dynamics. We note that evidence for autocorrelation in general was weaker than covariation so population simulation in this respect should be taken with caution. As expected, positive covariation and autocorrelation have a destabilizing effect on population dynamics, that is, both increase the temporal variance of the population size. These results are consistent with previous empirical studies showing that covariation among vital rates may contribute strongly to the temporal variation of the population growth rate (Coulson et al., 2005; Ezard et al., 2006). Some limits of the approach have to be kept in mind. Age‐dependent breeding probabilities were unknown empirically and have to be fixed in our population model. We have chosen a value such that the population remains stable on average. The use of different values resulting in increasing or decreasing populations led to weaker effects of covariation and autocorrelation on population dynamics, as discussed in Appendix S3. However, the effects of covariation and autocorrelation could also be underestimated in our study since vital rates are estimated with a non‐negligible error (Figure 1) that unavoidably lead to an underestimation of the covariation and autocorrelation estimate. Furthermore, covariation in other vital rates, like recruitment and adult breeding probability which are expected to vary similarly with food availability as productivity (Ezard et al., 2006; Laaksonen et al., 2004), has not been taken into account.

### 4.5 Estimation errors and inference

Most of the previous empirical studies estimating temporal correlation and autocorrelation in vital rates did not report errors around the mean of the estimates (e.g., Coulson et al., 2005; Davison et al., 2013; Morris et al., 2011; Sim et al., 2011). These studies used the estimated mean for inference without evaluating the statistical significance of these values. In the current study, we report 95% credible intervals for all estimates. These intervals were large and included 0 in all cases. This result is consistent with those from Gilljam et al. (2019) who stressed that uncertainty associated with the estimation of correlations is generally high and the type II error frequent. In our case, low precision is partly due to the length of the time series (15 years) and to the error propagation from the estimates of the vital rates to subsequent estimates of covariation and autocorrelation in the joint model. However, large credible intervals do not prohibit inference. Rather than making rigid conclusions ignoring uncertainty, we followed a balanced inference method weighing the evidence against uncertainty. The probability that the value is positive gives information to weight the confidence toward estimates. In this respect, positive covariation between survival and reproduction seems well supported and positive autocorrelation in survival is suggested but the evidence is weak.

### 4.6 Perspective

Until now, most population studies considered vital rates that fluctuate independently from each other. Yet, temporal covariation and autocorrelation appear to be structuring factors of population dynamics to a similar degree as age or sex. Several critical issues are still not well understood and need further empirical investigation. For example, which life‐history features and ecological factors make a species sensitive to survival‐reproduction covariation? How do individual traits like age and sex affect covariation and autocorrelation patterns? Does the demographic context determine which regulating factor, i.e. environmental condition, density dependence or trade‐offs, control covariation and autocorrelation patterns? A better understanding of these patterns would be particularly valuable for producing more reliable population forecasts. Indeed, population viability analyses are often applied to small populations for which limited demographic information is available. For those, correlation and autocorrelation structure are mostly unknown. Current recommendations are to examine a large variety of trait structures (Fieberg & Ellner, 2001), but uncertainty about which of them are most relevant remains high. In such a case, only a better understanding of the biological and ecological factors that drive correlation patterns both among and within vital rates in general could compensate for the lack of empirical data.

## CONFLICT OF INTEREST

None declared.

## AUTHOR CONTRIBUTIONS

All authors conceived the ideas and designed the methodology; SM, JJ, and JL coordinated data collection and management; RF analyzed the data; RF led the writing of the manuscript. All authors contributed to the drafts and gave final approval for publication.

## Data Availability

Dryad deposit: https://doi.org/10.5061/dryad.4152128.
