# Supplementary material for: Can temporal covariation and autocorrelation in demographic rates affect population dynamics in a raptor species?
Source: Ecol Evol. 2020 Feb 7;10(4):1959–70. doi: 10.1002/ece3.6027 (PMC7042680; doi:10.1002/ece3.6027)

Can temporal covariation and autocorrelation in demographic rates affect population dynamics in a raptor species?

Rémi Fay*^1^, Stephanie Michler^1^, Jacques Laesser^1^, Jacques Jeanmonod^1^ and Michael Schaub^1^

^1^ Swiss Ornithological Institute, Seerose 1, CH–6204 Sempach

*corresponding author: fay.remi@gmail.com

**Supplementary material**

**Appendix S1:** Multistate mark-recapture model and jags code to analyze capture-mark-recapture and recovery data.

True states:

1: juvenile alive, aluminum ring

2: adult alive, aluminum ring

3: adult alive physically captured and alphanumeric ring fixed,

4: adult alive resighted with alphanumeric ring

5: recently dead

6: long time dead

Transition matrix

| 0 | $S_{1}(1-a)$ | $S_{1}a$ | 0 | $1-S_{1}$ | 0 |
| --- | --- | --- | --- | --- | --- |
| 0 | $S_{2}(1-a)$ | $S_{2}a$ | 0 | $1-S_{2}$ | 0 |
| 0 | 0 | 0 | $S_{2}$ | $1-S_{2}$ | 0 |
| 0 | 0 | 0 | $S_{2}$ | $1-S_{2}$ | 0 |
| 0 | 0 | 0 | 0 | 0 | 1 |
| 0 | 0 | 0 | 0 | 0 | 1 |

t

Vector with state-specific recapture probabilities

| 0 | p | 1 | c | r | 0 |
| --- | --- | --- | --- | --- | --- |

t

Parameters:

$S_{1}$: Juvenile survival

$S_{2}$: Adult survival

$a$: Probability that an alphanumeric ring is fixed on a captured individual

p: Recapture probability of an adult with aluminum ring only

c: Resighting probability of an adult with both aluminum and an alphanumeric ring

r: Dead recovery probability

Jags Code

cat(file = "multistate.jags", "

model {

# Priors

for (t in 1:(n.occasions-1)){

sj[t] <- 1/(1+exp(-logit.sj[t]))

logit.sj[t] ~ dnorm(l.mean.sj, tau.sj)

sa[t] <- 1/(1+exp(-logit.sa[t]))

logit.sa[t] ~ dnorm(l.mean.sa, tau.sa)

a[t] <- 1/(1+exp(-logit.a[t]))

logit.a[t] ~ dnorm(l.mean.a, tau.a)

p[t] <- 1/(1+exp(-logit.p[t]))

logit.p[t] ~ dnorm(l.mean.p, tau.p)

c[t] <- 1/(1+exp(-logit.c[t]))

logit.c[t] ~ dnorm(l.mean.c, tau.c)

r[t] <- 1/(1+exp(-logit.r[t]))

logit.r[t] ~ dnorm(l.mean.r, tau.r)

}

l.mean.sj <- log(mean.sj/(1-mean.sj))

mean.sj ~ dunif(0, 1)

l.mean.sa <- log(mean.sa/(1-mean.sa))

mean.sa ~ dunif(0, 1)

l.mean.a <- log(mean.a/(1-mean.a))

mean.a ~ dunif(0, 1)

l.mean.p <- log(mean.p/(1-mean.p))

mean.p ~ dunif(0, 1)

l.mean.c <- log(mean.c/(1-mean.c))

mean.c ~ dunif(0, 1)

l.mean.r <- log(mean.r/(1-mean.r))

mean.r ~ dunif(0, 1)

tau.sj <- pow(sigma.sj, -2)

sigma.sj ~ dunif(0.001, 10)

tau.sa <- pow(sigma.sa, -2)

sigma.sa ~ dunif(0.001, 10)

tau.a <- pow(sigma.a, -2)

sigma.a ~ dunif(0.001, 10)

tau.p <- pow(sigma.p, -2)

sigma.p ~ dunif(0.001, 10)

tau.c <- pow(sigma.c, -2)

sigma.c ~ dunif(0.001, 10)

tau.r <- pow(sigma.r, -2)

sigma.r ~ dunif(0.001, 10)

# Define state-transition and observation probabilities

for (t in 1:(n.occasions-1)){

ps[1,t,1] <- 0

ps[1,t,2] <- sj[t]*(1-a[t])

ps[1,t,3] <- sj[t]*a[t]

ps[1,t,4] <- 0

ps[1,t,5] <- 1-sj[t]

ps[1,t,6] <- 0

ps[2,t,1] <- 0

ps[2,t,2] <- sa[t]*(1-a[t])

ps[2,t,3] <- sa[t]*a[t]

ps[2,t,4] <- 0

ps[2,t,5] <- 1-sa[t]

ps[2,t,6] <- 0

ps[3,t,1] <- 0

ps[3,t,2] <- 0

ps[3,t,3] <- 0

ps[3,t,4] <- sa[t]

ps[3,t,5] <- 1-sa[t]

ps[3,t,6] <- 0

ps[4,t,1] <- 0

ps[4,t,2] <- 0

ps[4,t,3] <- 0

ps[4,t,4] <- sa[t]

ps[4,t,5] <- 1-sa[t]

ps[4,t,6] <- 0

ps[5,t,1] <- 0

ps[5,t,2] <- 0

ps[5,t,3] <- 0

ps[5,t,4] <- 0

ps[5,t,5] <- 0

ps[5,t,6] <- 1

ps[6,t,1] <- 0

ps[6,t,2] <- 0

ps[6,t,3] <- 0

ps[6,t,4] <- 0

ps[6,t,5] <- 0

ps[6,t,6] <- 1

po[1,t,1] <- 0

po[1,t,2] <- p[t]

po[1,t,3] <- 1

po[1,t,4] <- c[t]

po[1,t,5] <- r[t]

po[1,t,6] <- 0

for (s in 2:ns){

for (u in 1:ns){

po[s,t,u] <- po[1,t,u]

} # u

} # s

for (s in 1:ns){

for (u in 1:ns){

qo[s,t,u] <- 1-po[s,t,u]

} # u

} # s

} # t

# Define the multinomial likelihood

for (t in 1:((n.occasions-1)*ns)){

marr[t,1:(n.occasions*ns-(ns-1))] ~ dmulti(pr[t, ], rel[t])

}

# Define the cell probabilities of the m-array

# Define matrix Q: product of probabilities of survival and non-capture

for (t in 1:(n.occasions-2)){

Q[(t-1)*ns+(1:ns), (t-1)*ns+(1:ns)] <- ones

for (j in (t+1):(n.occasions-1)){

Q[(t-1)*ns+(1:ns), (j-1)*ns+(1:ns)] <- Q[(t-1)*ns+(1:ns), (j-

2)*ns+(1:ns)] %*% (ps[,t,] * qo[,t,])

}

}

Q[(n.occasions-2)*ns+(1:ns), (n.occasions-2)*ns+(1:ns)] <- ones

# Define the cell probabilities of the multistate m-array

# The main diagonal

for (t in 1:(n.occasions-2)){

pr[(t-1)*ns+(1:ns),(t-1)*ns+(1:ns)] <- Q[(t-1)*ns+(1:ns), (t-

1)*ns+(1:ns)] %*% (ps[,t,] * po[,t,])

# Above main diagonal

for (j in (t+1):(n.occasions-1)){

pr[(t-1)*ns+(1:ns), (j-1)*ns+(1:ns)] <- Q[(t-1)*ns+(1:ns), (j-

1)*ns+(1:ns)] %*% (ps[,j,] * po[,j,])

}

}

pr[(n.occasions-2)*ns+(1:ns), (n.occasions-2)*ns+(1:ns)] <-

ps[,n.occasions-1,] * po[,n.occasions-1,]

# Below main diagonal

for (t in 2:(n.occasions-1)){

for (j in 1:(t-1)){

pr[(t-1)*ns+(1:ns),(j-1)*ns+(1:ns)] <- zero

} #j

} #t

# Last column: probability of non-recapture

for (t in 1:((n.occasions-1)*ns)){

pr[t,(n.occasions*ns-(ns-1))] <- 1-sum(pr[t,1:((n.occasions-

1)*ns)])

} #t

}

")

# Number of state

ns <- 6

# Bundle data

jags.data <- list(marr = ms.arr, n.occasions = ncol(ch.new), rel = rowSums(ms.arr), ns = ns, zero = matrix(0, ncol = ns, nrow = ns), ones = diag(ns))

# Initial values

inits <- function(){list()}

parameters <- c("sj", "sa", "mean.sj", "mean.sa", "mean.a", "mean.p", "mean.c", "mean.r", "sigma.sj", "sigma.sa", "sigma.a", "sigma.p", "sigma.c", "sigma.r")

# MCMC settings

ni <- 50000

nt <- 10

nb <- 20000

nc <- 3

# Call JAGS from R

out1 <- jags(jags.data, inits, parameters, "multistate.jags", n.chains = nc, n.thin = nt, n.iter = ni, n.burnin = nb, parallel = TRUE, n.adapt = 5000)

**Appendix S2**: Script used to fit the multivariate normal model

# Priors for the multivariate normal distribution

for (t in 1:(n.occasions-1)){

cov.traits[t,1:3] ~ dmnorm.vcov(l.mu[1:3], prec.Sigma[1:3,1:3])

}

l.mu[1] <- log(mu.sa / (1-mu.sa))

mu.sa ~ dunif(0, 1)

l.mu[2] <- log(mu.sj / (1-mu.sj))

mu.sj ~ dunif(0, 1)

l.mu[3] <- log(mu.prod)

mu.prod ~ dunif(0.01, 10)

## Matrice of variance covariance

prec.Sigma[1,1] <- pow(sigma.sa, 2)

sigma.sa ~ dunif(0.01,5)

prec.Sigma[2,2] <- pow(sigma.sj, 2)

sigma.sj ~ dunif(0.01,5)

prec.Sigma[3,3] <- pow(sigma.prod, 2)

sigma.prod ~ dunif(0.01,10)

prec.Sigma[1,2] <- cov1

prec.Sigma[2,1] <- cov1

prec.Sigma[1,3] <- cov2

prec.Sigma[3,1] <- cov2

prec.Sigma[2,3] <- cov3

prec.Sigma[3,2] <- cov3

cov1 <- cor.sj.sa * sigma.sj * sigma.a

cov2 <- cor.sj.prod * sigma.sj * sigma.prod

cov3 <- cor.sa.prod * sigma.sa * sigma.prod

cor.sj.sa ~ dunif(-1,1)

cor.sj.prod ~ dunif(-1,1)

#For the third correlation we applied a constraint on the uniform distribution to ensure that the variance-covariance matrix is positively defined (see equation 1 in Budden et al. 2006 Applied Mathematics)

Lower <- cor.sj.sa*cor.sj.prod-sqrt((1-cor.sj.sa^2)*(1-cor.sj.prod^2))

Upper <- cor.sj.sa*cor.sj.prod+sqrt((1-cor.sj.sa^2)*(1-cor.sj.prod^2))

cor.sa.prod ~ dunif(Lower,Upper)

# Priors and constraints for others parameters

for (t in 1:(n.occasions-1)){

sj[t] <- 1/(1+exp(-cov.traits[t,2]))

sa[t] <- 1/(1+exp(-cov.traits[t,1]))

a[t] <- 1/(1+exp(-logit.a[t]))

logit.a[t] ~ dnorm(l.mean.a, tau.a)

p[t] <- 1/(1+exp(-logit.p[t]))

logit.p[t] ~ dnorm(l.mean.p, tau.p)

c[t] <- 1/(1+exp(-logit.c[t]))

logit.c[t] ~ dnorm(l.mean.c, tau.c)

r[t] <- 1/(1+exp(-logit.r[t]))

logit.r[t] ~ dnorm(l.mean.r, tau.r)

prod[t] <- exp(cov.traits[t,3])

}

l.mean.a <- log(mean.a / (1-mean.a))

mean.a ~ dunif(0, 1)

l.mean.p <- log(mean.p / (1-mean.p))

mean.p ~ dunif(0, 1)

l.mean.c <- log(mean.c / (1-mean.c))

mean.c ~ dunif(0, 1)

l.mean.r <- log(mean.r / (1-mean.r))

mean.r ~ dunif(0, 1)

tau.a <- pow(sigma.a, -2)

sigma.a ~ dunif(0.001, 10)

tau.p <- pow(sigma.p, -2)

sigma.p ~ dunif(0.001, 10)

tau.c <- pow(sigma.c, -2)

sigma.c ~ dunif(0.001, 10)

tau.r <- pow(sigma.r, -2)

sigma.r ~ dunif(0.001, 10)

tau.obs <- pow(sigma.obs, -2)

sigma.obs ~ dunif(0.001, 20)

# Define state-transition and observation probabilities

for (t in 1:(n.occasions-1)){

ps[1,t,1] <- 0

ps[1,t,2] <- sj[t]*(1-a[t])

ps[1,t,3] <- sj[t]*a[t]

ps[1,t,4] <- 0

ps[1,t,5] <- 1-sj[t]

ps[1,t,6] <- 0

ps[2,t,1] <- 0

ps[2,t,2] <- sa[t]*(1-a[t])

ps[2,t,3] <- sa[t]*a[t]

ps[2,t,4] <- 0

ps[2,t,5] <- 1-sa[t]

ps[2,t,6] <- 0

ps[3,t,1] <- 0

ps[3,t,2] <- 0

ps[3,t,3] <- 0

ps[3,t,4] <- sa[t]

ps[3,t,5] <- 1-sa[t]

ps[3,t,6] <- 0

ps[4,t,1] <- 0

ps[4,t,2] <- 0

ps[4,t,3] <- 0

ps[4,t,4] <- sa[t]

ps[4,t,5] <- 1-sa[t]

ps[4,t,6] <- 0

ps[5,t,1] <- 0

ps[5,t,2] <- 0

ps[5,t,3] <- 0

ps[5,t,4] <- 0

ps[5,t,5] <- 0

ps[5,t,6] <- 1

ps[6,t,1] <- 0

ps[6,t,2] <- 0

ps[6,t,3] <- 0

ps[6,t,4] <- 0

ps[6,t,5] <- 0

ps[6,t,6] <- 1

po[1,t,1] <- 0

po[1,t,2] <- p[t]

po[1,t,3] <- 1

po[1,t,4] <- c[t]

po[1,t,5] <- r[t]

po[1,t,6] <- 0

for (s in 2:ns){

for (u in 1:ns){

po[s,t,u] <- po[1,t,u]

} # u

} # s

for (s in 1:ns){

for (u in 1:ns){

qo[s,t,u] <- 1-po[s,t,u]

} # u

} # s

} # t

# Define the multinomial likelihood

for (t in 1:((n.occasions-1)*ns)){

marr[t,1:(n.occasions*ns-(ns-1))] ~ dmulti(pr[t, ], rel[t])

}

# Define the cell probabilities of the m-array

# Define matrix Q: product of probabilities of survival and non-capture

for (t in 1:(n.occasions-2)){

Q[(t-1)*ns+(1:ns), (t-1)*ns+(1:ns)] <- ones

for (j in (t+1):(n.occasions-1)){

Q[(t-1)*ns+(1:ns), (j-1)*ns+(1:ns)] <- Q[(t-1)*ns+(1:ns), (j-

2)*ns+(1:ns)] %*% (ps[,t,] * qo[,t,])

}

}

Q[(n.occasions-2)*ns+(1:ns), (n.occasions-2)*ns+(1:ns)] <- ones

# Define the cell probabilities of the multistate m-array

# The main diagonal

for (t in 1:(n.occasions-2)){

pr[(t-1)*ns+(1:ns),(t-1)*ns+(1:ns)] <- Q[(t-1)*ns+(1:ns), (t-

1)*ns+(1:ns)] %*% (ps[,t,] * po[,t,])

# Above main diagonal

for (j in (t+1):(n.occasions-1)){

pr[(t-1)*ns+(1:ns), (j-1)*ns+(1:ns)] <- Q[(t-1)*ns+(1:ns), (j-

1)*ns+(1:ns)] %*% (ps[,j,] * po[,j,])

}

}

pr[(n.occasions-2)*ns+(1:ns), (n.occasions-2)*ns+(1:ns)] <-

ps[,n.occasions-1,] * po[,n.occasions-1,]

# Below main diagonal

for (t in 2:(n.occasions-1)){

for (j in 1:(t-1)){

pr[(t-1)*ns+(1:ns),(j-1)*ns+(1:ns)] <- zero

} #j

} #t

# Last column: probability of non-recapture

for (t in 1:((n.occasions-1)*ns)){

pr[t,(n.occasions*ns-(ns-1))] <- 1-sum(pr[t,1:((n.occasions-

1)*ns)])

} #t

# Regression model for productivity data

for (t in 1:(n.occasions-1)){

for (i in 1: n.product[t]){

n.chick[i,t] ~ dnorm(prod[t], tau.obs)

}

}

}

")

# Number of states

ns <- 6

# Bundle data

jags.data <- list(marr = ms.arr, n.occasions = ncol(ch.new), rel = rowSums(ms.arr), ns = ns, zero = matrix(0, ncol = ns, nrow = ns), ones = diag(ns), n.chick = PROD, n.product = n.product)

# Initial values

inits <- function(){list()}

parameters <- c("sj", "sa", “prod”, "mu.sj", "mu.sa", “mu.prod”, "mean.a", "mean.p", "mean.c", "mean.r", "sigma.sj", "sigma.sa", “sigma.prod”, "sigma.a", "sigma.p", "sigma.c", "sigma.r", “sigma.obs”)

# MCMC settings

ni <- 50000

nt <- 10

nb <- 20000

nc <- 3

# Call JAGS from R

Out2 <- jags(jags.data, inits, parameters, "multistate.jags", n.chains = nc, n.thin = nt, n.iter = ni, n.burnin = nb, parallel = TRUE, n.adapt = 5000)

**Appendix S3**: Population simulation

We assessed the potential effect of the estimated covariation and autocorrelation in vital rates using population simulation. We did not include the covariation between juvenile and adult survival as well as the temporal autocorrelation in productivity because mean estimates were close to 0 for these parameters. We distinguished the minimal from the maximal estimates of covariation and autocorrelation that are given by the different models. The minimal estimate of covariation originates from the multivariate normal model and the minimal estimate of autocorrelation from the model estimating the vitals rate with a fixed time effect (see methods section). The maximal estimate of covariation originates from the regression approach and the maximal estimate of autocorrelation from the residual decomposition.

To obtain year specific vital rates, we first simulated productivity values from a normal distribution based on the estimated mean ($\hat{\delta}$) and variance ($\hat{\sigma}^{2}$), thus $\delta_{t} \sim N\left( \hat{\delta},\hat{\sigma}^{2} \right)$. Then we generated correlated survival values using linear regressions:

${logit(S}_{t})$ = $\beta_{0}$ + $\beta_{1}$ * $\delta_{t}$+ $\varepsilon_{t}$

For the maximal covariation estimate we used the slope $\beta_{1}$ estimated by the regression approach. For the minimal covariation estimates by the multivariate normal distribution, we computed the corresponding slope from the correlation estimate using the following formula:

$slope=\frac{\sqrt{\sum_{i=1}^{n} {({logit(s}_{i})- logit(\bar{s}))}^{2}}}{\sqrt{\sum_{i=1}^{n} {(\delta_{i}- \bar{\delta})}^{2}}}$ * r where n is the number of years for which survival (s) and productivity ($\delta$) are estimated and r is the correlation coefficient.

Temporal autocorrelation was introduced using an autocorrelated noise. Once the annual vital rates have been simulated with the desired level of covariation and autocorrelation, we just reordered the set of these values to obtain vital rates with only correlation, only autocorrelation or neither covariation nor autocorrelation. For instance by reordering all vital rates with the same arbitrary rank, we obtain vital rates which are still correlated but which are not autocorrelated anymore. This procedure ensures that the comparison between the different scenarios is not affected by other factors than the presence or absence of covariation and autocorrelation.

Our population model includes the parameter Ω that captures vital rates that we have not estimated, i.e. recruitment and adult breeding probability. Although unknown, the value of this parameter is necessarily between 0 and 1 since it is a probability. We fixed Ω in the population simulation to the value needed to obtain a stable population. Alternative values of Ω that result in a positive or negative population growth rate had a marginal effect on the results. The effect of covariation and autocorrelation on the mean population growth rate remains negligible whatever the value of Ω. However, the effect of covariation and autocorrelation on the variability of the population size changes quantitatively, but not qualitatively, when Ω varies. When Ω is close to 0 or 1, the influence of covariation and temporal autocorrelation in vital rates on the variability of population size decreases strongly being almost negligible. This is mainly an undue artefact due to the high effect of Ω on the population growth rate. In reality, the vital rates captured in the parameter Ω are not expected to have a higher impact on kestrel population dynamics than survival and productivity. Furthermore the vital rates gathered in parameter Ω are expected to be variable and positively correlated with, at least, productivity (Laaksonen et al. 2004; Ezard et al. 2006). Thus, using a constant Ω value that affects the population growth rate likely leads to an underestimation of the potential effects of covariation and autocorrelation in survival and productivity.

**Appendix S4**: Explanation of the outlier

Grass land is the key foraging habitat for kestrels in our study area. Grass land is intensively exploited by farmers with regular mowing from April to September. Frequent mowing of grassland is expected to be favorable for foraging kestrels as it increases the accessibility to voles (Casagrande et al. 2008). Kestrels preferable hunt in grassland with less than 20 cm vegetation height and thus show strong preference for freshly mown surfaces (Aschwanden et al. 2005). In 2013, the frequency of mowing has been strongly shifted compared to the usual mowing regime. The period from April to June, corresponding to the laying, brooding, hatching and rearing stage of kestrels, was exceptionally wet and cold (Fig. S2), such that mowing occurred less frequently than normal. The vegetation height therefore remained high during much of the breeding season hampering easy prey accessibility and resulting in low reproductive success. However during the fledging period (July-August), weather conditions became favorable for mowing with a lot of sunshine and little precipitation (Fig. S2). Thus, in contrast to the first period prey accessibility in the second period was much improved, likely resulting in high juvenile survival. This mechanism likely explains why there was no positive relationship between breeding success and juvenile survival in 2013. 2013 could be considered as an outlier because the process generating reproduction survival covariation behaves unusually during this year.

References

Aschwanden, J., Birrer, S., & Jenni, L. (2005). Are ecological compensation areas attractive hunting sites for common kestrels (Falco tinnunculus) and long-eared owls (Asio otus)? *Journal of Ornithology*, *146*(3), 279–286. doi:10.1007/s10336-005-0090-9

Casagrande, S., L. Nieder, E. Di Minin, I. La Fata, and D. Csermely. 2008. Habitat utilization and prey selection of the kestrel Falco tinnunculus in relation to small mammal abundance. Italian journal of zoology 75:401–409.

Figure S1: Map of Switzerland with the study area in gray and the sub area where prey remains have been recorded (black circle).


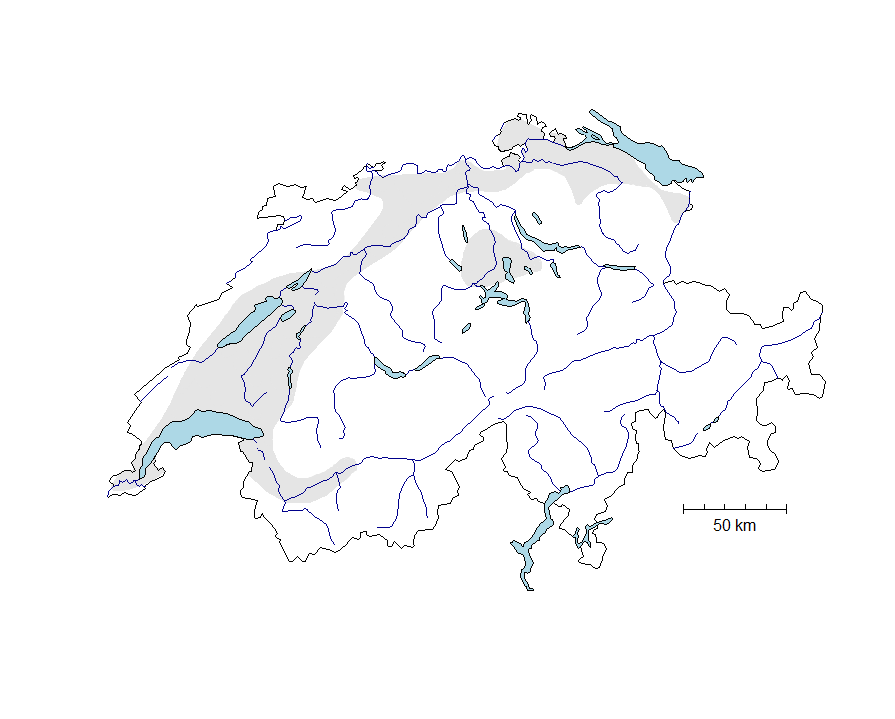


Figure S2: Yearly average sunshine duration, precipitation and temperature during a) April-May-June and b) July-August in Switzerland over the study duration.


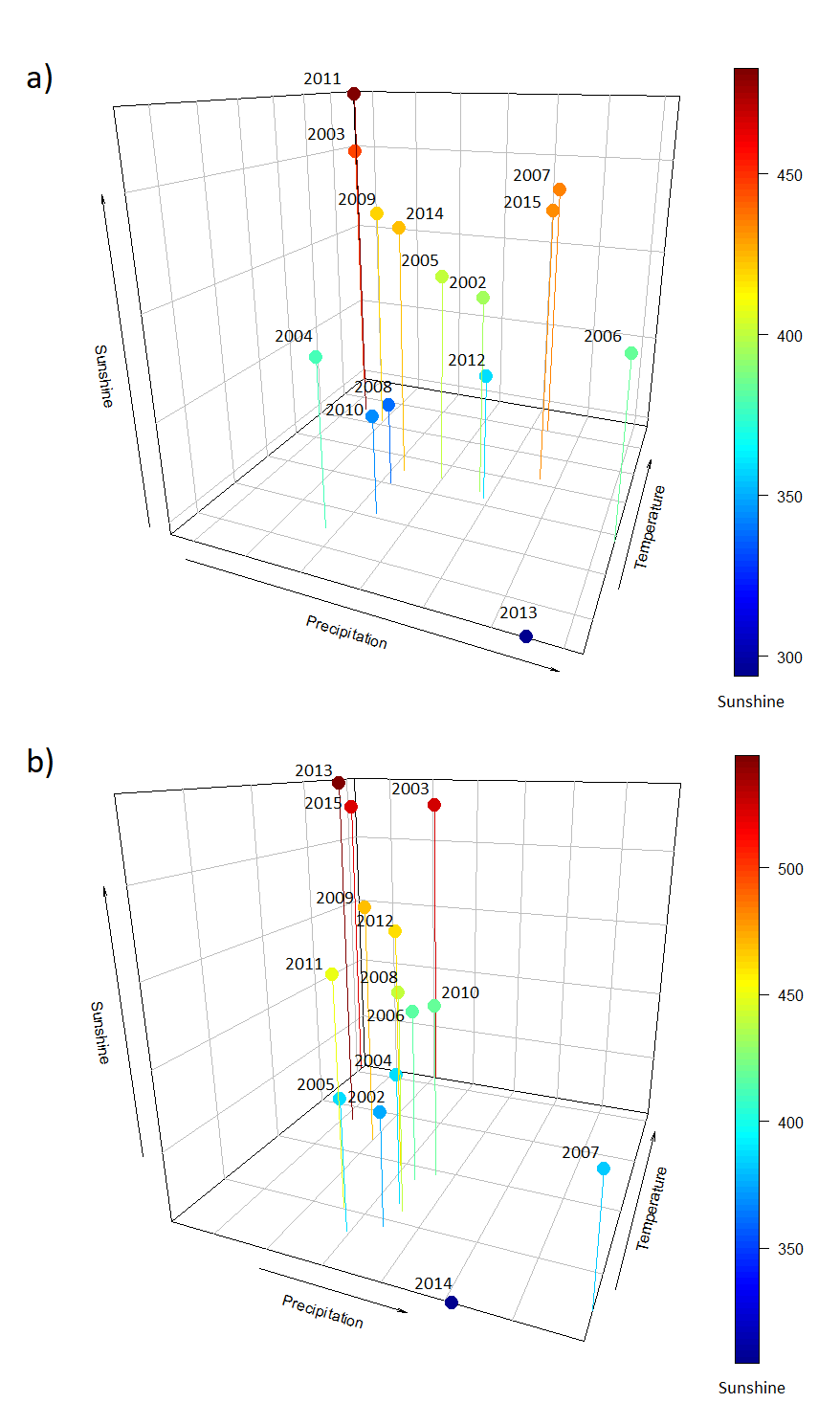


Figure S3: Investigating the robustness of the autocorrelation estimates

For a given trait, one year lag autocorrelation is the average correlation of this trait with itself between two consecutive years. To investigate the robustness of the results, we re-estimated the autocorrelation after ignoring each pair of years one by one and computed the difference between the autocorrelation obtained considering all the years and the new estimate. Results show that autocorrelation estimates for juvenile and adult survival are unchanged whatever the pair of years removed. However the autocorrelation estimate for productivity is strongly affected when we ignore the correlation between years 2012 and 2013. The negative autocorrelation estimate reflects only the extreme variation of productivity between these two years. In other words, the negative average autocorrelation value for productivity does not reflect the usual year to year correlation which is confirmed by a graphical examination (Fig. S4).


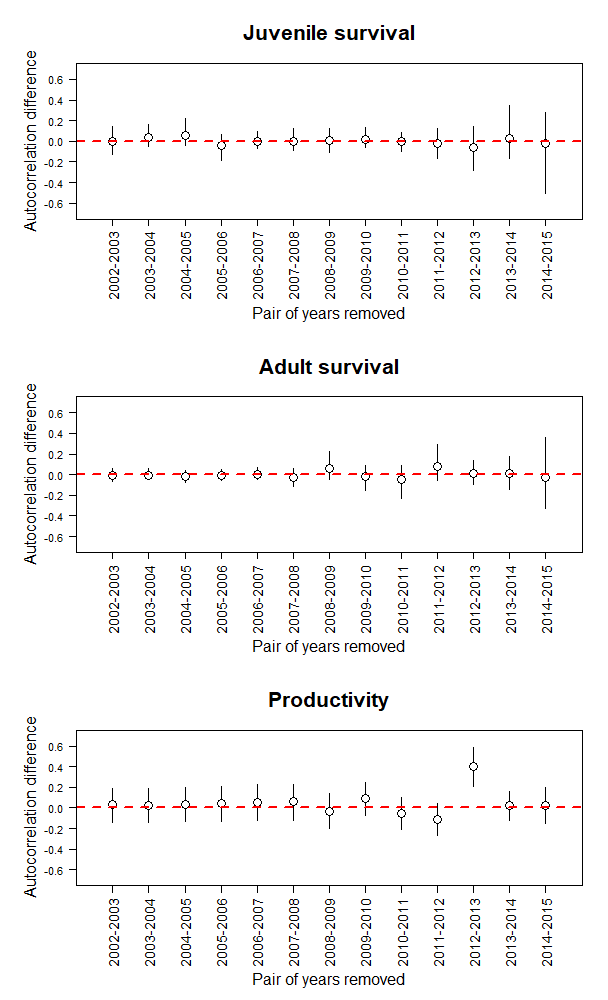


Figure S4: Graphical examination of autocorrelation patterns.

The graphical examination of autocorrelation in vital rates suggests no clear trend for productivity. The negative autocorrelation suggested by the statistical analyses seems spurious being driven by one extreme point (pair 2012-2013 in red). The sign of the of autocorrelation estimate for productivity changes from negative to positive when the red point is ignored.


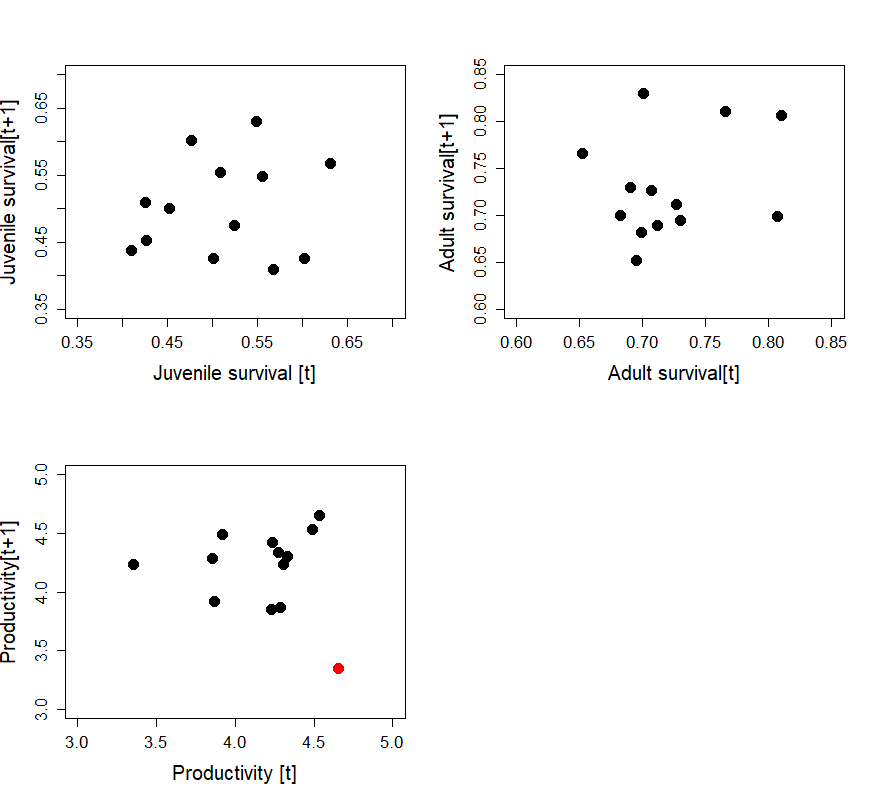

Supplement: Supplementary file 1 [file ECE3-10-1959-s001.docx]
